# Freezing of gait in Parkinson’s disease is associated with the microstructural and functional changes of globus pallidus internus

**DOI:** 10.3389/fnagi.2022.975068

**Published:** 2022-08-18

**Authors:** Wenyi Kou, Xuemei Wang, Yuanchu Zheng, Jiajia Zhao, Huihui Cai, Huimin Chen, Binbin Sui, Tao Feng

**Affiliations:** ^1^Center for Movement Disorders, Department of Neurology, Beijing Tiantan Hospital, Capital Medical University, Beijing, China; ^2^Department of Neurology, Beijing Hospital, National Center of Gerontology, Institute of Geriatric Medicine, Chinese Academy of Medical Sciences, Beijing, China; ^3^Tiantan Neuroimaging Center for Excellence, Beijing Tiantan Hospital, Capital Medical University, Beijing, China; ^4^China National Clinical Research Center for Neurological Diseases, Beijing, China

**Keywords:** Parkinson’s disease, freezing of gait, diffusion tensor imaging, resting-state fMRI, microstructure, globus pallidus internus

## Abstract

**Background:**

Freezing of gait (FOG) is a common motor symptom in advanced Parkinson’s disease (PD). However, the pathophysiology mechanism of FOG is not fully understood. The purpose of this study was to investigate microstructural abnormalities in subcortical gray matter and alterations in functional connectivity of the nuclei with microstructural changes. In addition, the correlations between these microstructural and functional changes and the severity of FOG were measured.

**Materials and methods:**

Twenty-four patients with FOG (PD-FOG), 22 PD patients without FOG (PD-nFOG), and 27 healthy controls (HC) were recruited. FOG Questionnaire (FOGQ) and Gait and Falling Questionnaire (GFQ) were assessed, and Timed Up and Go (TUG) tests were performed in PD-FOG patients. All subjects underwent diffusion tensor imaging (DTI) and resting-state functional MRI scanning. The DTI measures, including fractional anisotropy (FA), mean diffusivity (MD), radial diffusivity (RD), and axial diffusivity (AD), were extracted and measured from basal ganglia, thalamus, and substantia nigra. The nuclei with microstructural alterations were selected as seed regions to perform the seed-based resting-state functional connectivity.

**Results:**

The MD and RD values of the right globus pallidus internus (GPi) were significantly higher in patients with PD-FOG compared with PD-nFOG patients and HC. In PD-FOG patients, the MD and RD values of the right GPi were significantly correlated with the time of the TUG test in both ON and OFF states. The MD values were also correlated with the GFQ scores in PD-FOG patients. Resting-state functional connectivity between the right GPi and left middle occipital gyri decreased significantly in PD-FOG patients compared to PD-nFOG patients, and was negatively correlated with GFQ scores as well as the time of ON state TUG in PD-FOG patients.

**Conclusion:**

Microstructural alterations in the right GPi and functional connectivity between the right GPi and visual cortex may be associated with the pathophysiological mechanisms of FOG in PD patients.

## Introduction

Freezing of gait (FOG), a common motor symptom in Parkinson’s disease (PD), is described as a momentary, episodic absence or marked reduction of forwarding movement of the feet despite the intention to walk (Giladi and Nieuwboer, 2008; Nutt et al., 2011). It is a debilitating symptom that severely affects the quality of life and increases the risk of falls (Bloem et al., 2004; Moore et al., 2007). The prevalence of FOG in patients with early PD is 21–27% (Giladi et al., 2001; Tan et al., 2011). While in the later stages, this number increases to 80% (Hely et al., 2008; Tan et al., 2011). However, the pathophysiological mechanisms of FOG are not fully understood.

Multiple brain regions in the basal ganglia circuit have been suggested to be associated with FOG in PD (Lewis and Barker, 2009; Shine et al., 2013a). Of these, the output nuclei of the basal ganglia, globus pallidus internus (GPi), play an important role in the pathophysiological mechanisms of FOG (Lewis and Shine, 2016). It has been shown that beta desynchronization in the GPi is detected from rest to gait initiation in PD patients with FOG (PD-FOG) (Molina et al., 2020). Task-based functional MRI has observed a significant decrease of brain activity in bilateral GPi during episodes of freezing of gait (Shine et al., 2013a). In resting-state functional MRI, the amplitude of low-frequency fluctuation (ALFF) in bilateral globus pallidus was positively correlated with FOG severity (Mi et al., 2017).

Despite several studies confirming the presence of functional alterations of GPi in PD-FOG, a limited number of studies have concentrated on the microstructural differences between PD-FOG and PD patients without FOG (PD-nFOG). The microstructure can be sensitively quantified using diffusion tensor imaging (DTI), by detecting altered diffusion patterns of water molecules in brain tissue (Mori and Zhang, 2006). Higher mean diffusivity (MD) value was reported in basal ganglia in PD-FOG than PD-nFOG group, but the microstructure of certain nuclei in the basal ganglia has not been assessed (Youn et al., 2015).

Several nuclei have been used as predefined seeds, for instance, pedunculopontine, thalamus, and dentate nucleus for seed-based resting-state functional connectivity (rs-FC) analysis, and observed that their FCs were altered in PD-FOG patients (Wang et al., 2016; Potvin-Desrochers et al., 2019; Bharti et al., 2020). Network-level analysis showed inter-network FC alterations, such as, sensorimotor network, default mode network, frontoparietal network, and basal ganglia network in PD-FOG patients (Canu et al., 2015; Bharti et al., 2020). In this research, nuclei with microstructural alternations were further used as seed regions for investigating seed-based rs-FC alterations.

In the current study, we used the region of interest (ROI)-based DTI technique to explore the microstructure of nuclei in the basal ganglia circuit, including the bilateral caudate, putamen, GPi, globus pallidus externa (GPe), thalamus, and substantia nigra (SN) (An illustration of chosen ROIs is displayed in Supplementary Figure 1). To further investigate PD-FOG associated functional network alterations, nuclei with microstructural changes were used as seeds for resting-state fMRI analysis. In addition, a correlation analysis was performed of the altered microstructure and functional connectivity with the severity of FOG. The current study will help to understand the pathogenesis of FOG in PD.

## Materials and methods

### Participants

Forty-six right-handed patients diagnosed with idiopathic PD and 27 healthy controls (HC) were recruited from the Department of Neurology, Beijing Tiantan Hospital, Capital Medical University from April 2021 to December 2021. The diagnosis of PD was according to the Movement Disorder Society Clinical Diagnostic Criteria for PD (Postuma et al., 2015). Twenty-four patients (13 female, mean age 62.6 ± 9.74) who fulfilled the following conditions were categorized in the PD-FOG group: (a) FOG Questionnaire (FOGQ) item 3 score ≥ 1 (Bloem et al., 2016), and (b) FOG were observed by experienced movement disorders neurologists (Sunwoo et al., 2013). Twenty-two PD patients (11 female, mean age 63.8 ± 7.48) who did not fulfill the above criteria were included in the PD-nFOG group. Patients with a diagnosis of atypical parkinsonism were excluded. Exclusion criteria for all subjects included age < 18, Mini-mental State Examination (MMSE) score < 24, severe head tremor, deep brain stimulation implantation, with any disorders affecting gait or balance other than PD, or any contraindications for MRI scans.

Demographic details including age, gender, and educational years were obtained. The MMSE and Frontal Assessment Battery (FAB) (Dubois et al., 2000) were acquired to assess global cognitive function and executive function respectively. Hamilton Anxiety Scale (HAMA) (Hamilton, 1959), Hamilton Depression Scale (HAMD) (Hamilton, 1960), and Starkstein Apathy Scale (SAS) (Starkstein et al., 1992) scores were also collected. Disease severity was measured by the Movement Disorder Society Unified Parkinson’s Disease Rating Scale (MDS-UPDRS) part III (Goetz et al., 2008) and Hoehn and Yahr (H-Y) stage (Hoehn and Yahr, 1967) in the OFF medication state, after a minimum of 12 h withdrawal of all anti-PD medications. The accepted calculation protocol was used to compute levodopa equivalent daily dose (LEDD) (Tomlinson et al., 2010; Table 1). This study was approved by the Ethics Committee of Beijing Tiantan Hospital, Capital Medical University. All participants signed informed consent forms.

**TABLE 1:** Demographic and clinical characteristics.

	PD-FOG	PD-nFOG	HC	*p*
Age	62.6 ± 9.74	63.8 ± 7.48	63.8 ± 5.72	0.838
Gender (male/female)	11/13	11/11	14/13	0.909
Educational years	9.3 ± 3.90	10.7 ± 4.56	10.96 ± 2.59	0.239
Disease duration	5.5 (4.25∼8.0)	5.5 (4.0∼10.0)		0.991
H-Y stage	3.0 (3.0∼4.0)	3.0 (2.4∼3.0)		0.066
MDS-UPDRS-III	39.71 ± 12.87	39.0 ± 14.41		0.861
LEDD, mg/d	900.0 (680.0∼1187.5)	880.125 (575.0∼1146.0)		0.674
MMSE	28 (26.25∼29)	28 (25.75∼29.25)	29 (28∼30)	0.085
FAB	16.0 (15.0∼17.0)	16.0 (14.0∼18.0)		0.759
HAMA	7.7 ± 4.71	8.4 ± 4.03		0.533
HAMD	6.0 (4.0∼13.0)	5.0 (3.5∼9.0)		0.26
SAS	9.23 ± 7.19	11.9 ± 9.44		0.134

Data are presented as mean ± SD for normal distribution and median (upper quartile∼lower quartile) for non-normal distribution. H-Y stage, Hoehn-Yahr stage; MDS-UPDRS, movement disorder society unified Parkinson’s disease rating scale; LEDD, levodopa equivalent daily dose; MMSE, mini-mental state examination; FAB, frontal assessment battery; HAMA, Hamilton anxiety scale; HAMD, Hamilton depression scale; SAS, Starkstein apathy scale.

### Assessment of freezing of gait

The severity of FOG was evaluated using the FOGQ (Bloem et al., 2016) and Gait and Falling Questionnaire (GFQ) (Giladi et al., 2000). PD-FOG patients also performed the Timed Up and Go (TUG) test to assess gait. PD-FOG patients were required to stand up from a standard chair, walk three meters at a comfortable and safe speed, turn, walk back to the chair, and sit down. Each subject completed the TUG test twice in the OFF and ON states respectively. The test process was recorded by the camera. Two observers timed the performance according to the video, and the average duration was calculated (Podsiadlo and Richardson, 1991).

### Magnetic resonance imaging data acquisition

Neuroimaging was acquired on a 3-Tesla magnetic resonance (MR) scanner (Signa Premier, GE Healthcare, Milwaukee) using a 48-channel-phased array head coil. Scanning was performed during the OFF state for the patients. All subjects were instructed to keep their heads still and eyes open during scanning, but not to think or fall asleep.

Magnetic resonance protocol included the following sequences: 3D T1-weighted Magnetization Prepared Rapid Gradient Echo (MPRAGE) sequence [echo time (TE) = 2.2 ms, repetition time (TR) = 1952 ms, voxel size = 1.0 mm × 1.0 mm × 1.0 mm, slice space = 0.5 mm, matrix size = 256 × 256, slice number = 376]; DTI sequence (TE = 70 ms, TR = 3,236 ms, voxel size = 2.0 mm × 2.0 mm × 2.0 mm, matrix size = 104 × 104, slice number = 78, 64 diffusion-sensitizing gradients at a b-value of 1,000 s/mm^2^ and three b = 0 images); T2*-weighted blood oxygen level-dependent (BOLD) sequence was carried out for the resting state measurement (TE = 39 ms, TR = 1,000 ms, voxel size = 2.4 mm × 2.4 mm × 2.4 mm, matrix size = 86 × 86, 65 axial slices, 330 brain volumes). Both DTI and BOLD sequences were carried-out with the multiband accelerated echo planar imaging (EPI), with the new ARC acquisition pattern.

### Magnetic resonance imaging data processing

#### Diffusion tensor imaging

FMRIB Software Library tools^1^ was used to process DTI Imaging. The FMRIB’s Diffusion Toolbox was used to eliminate the eddy current distortions and head movements from the source diffusion data after it was translated to NIFTI format. The voxel-wise maps of fractional anisotropy (FA), mean diffusivity (MD), radial diffusivity (RD), and axial diffusivity (AD) were generated by applying the *dtifit* tool.

We extracted the ROI masks of the bilateral caudate, putamen, and thalamus from the Harvard-Oxford probabilistic atlas (Behrens et al., 2007). As the Harvard-Oxford atlas lacks labels for bilateral GPi, GPe, and SN, the Amsterdam Ultra-high field probabilistic atlas (Alkemade et al., 2020) was used to extract those masks. The probabilistic masks for each nucleus were thresholded to a probability of 80% and resliced to 2 × 2 × 2mm^3^ (Supplementary Figure 1).

The T1-image was subjected to brain extraction using *bet* and resliced to 2 × 2 × 2mm^3^. Each subject’s DTI maps were co-registered to the subject’s T1 space using the *flirt* tool, and the inverse transformation was obtained. The *flirt* and *fnirt* tools were used to normalize the subject’s T1 image to Montreal Neurological Institute (MNI) 152 standard space, and the *invwarp* tool was used to calculate the inverse transformation of T1 maps to standard space. Both inverse transformations were combined by using the *convertwarp* tool. For each participant, the *applywarp* tool was further used to co-register standard space ROI masks to the subject space. To verify the accuracy of placement, the ROI masks in the subject’s space were overlayed on each subject’s DTI map. Two PD-FOG patients were removed for poor registration. The mean MD, RD, AD, and FA values within the ROIs were obtained by the *fslstats* tool (Nagae et al., 2016).

#### Resting-state functional magnetic resonance imaging

Resting-state functional MRI (rs-fMRI) datasets were pre-processed using RESTplusV1.2^2^ and SPM12^3^ on the MATLAB R2013b platform. DICOM data were converted to NIFTI format. The first ten time points were removed. The remaining images were corrected for slice timing and realigned to the first volume for head motion correction. The head movement standard was set to less than 3 mm (translation) or 3°(rotation). Data from one HC subject and two PD-FOG subjects were excluded from the analysis due to excessive head movement. The images were normalized to the MNI152 standard space of 3 × 3 × 3 mm^3^. The effect of six head movement measures, as well as white matter and cerebrospinal fluid signals, was removed using linear regression. Then the images were spatially smoothed using a Gaussian kernel with a 6-mm full width at half maximum. Next, the data were linearly detrended and temporally bandpass filtered at a low frequency (0.01–0.08 Hz). Nuclei with microstructural alterations in the PD-FOG group compared with both the PD-nFOG and HC groups were used as seed regions. The seed-based resting-state functional connectivity (rs-FC) was performed using Pearson correlations. Then the correlation coefficients were calculated by Fisher’s Z transformation.

### Statistical analysis

Statistical analyses of demographic, clinical, and gait assessments were performed in SPSS25. The significant level was set to *p* < 0.05. Shapiro-Wilk test was performed for demographic and clinical data distribution. The detailed *p*-values for the Shapiro-Wilk test are shown in Supplementary Table 1. Gender differences among PD-FOG, PD-nFOG, and HC groups were assessed by the Chi-square test. Independent two-sample t-test was performed to evaluate the differences in MDS-UPDRS-III, HAMA, and SAS scores between PD-FOG and PD-nFOG patients. Differences in disease duration, H-Y stage, HAMD, and FAB were assessed by the Mann-Whitney U test. One-way analysis of variance (ANOVA) test was performed to evaluate the differences in age, years of education, and DTI measures (MD, RD, AD, FA) among the three groups. The non-parametric Kruskal-Wallis H test was performed to determine the differences in MMSE. A significant result was followed by a pairwise Bonferroni test to assess differences between the groups. Correlations between imaging results and FOGQ as well as GFQ scores were calculated using Pearson correlation. DTI measures and rs-FC correlations with TUG tests were calculated using the Spearman correlation.

Statistical analysis of the functional MRI data was performed using SPM12. Voxel-wise differences among PD-FOG, PD-nFOG, and HC groups were carried out using one-way ANOVA (voxel threshold *p* < 0.01, clusters AlphaSim corrected *p* < 0.05). To determine between-group rs-FC differences, independent two-sample *t*-test was further performed within a mask showing significant differences among the three groups (voxel threshold *p* < 0.01, clusters AlphaSim corrected *p* < 0.01).

## Results

### Demographic information

A total of 73 subjects were included in this study: 24 in the PD-FOG group (11 males and 13 females, 62.6 ± 9.74 years old), 22 in the PD-nFOG group (11 males and 11 females, 63.8 ± 7.48 years old), and 27 in the HC group (14 males and 13 females, 63.8 ± 5.72 years old). There was no significant difference in age, gender, educational years, and MMSE score among the three groups (Table 1). No significant differences were seen between the PD-FOG group and the PD-nFOG group in the disease duration, H-Y stage, MDS-UPDRS-III score, LEDD, FAB, HAMA, HAMD, and SAS scores (Table 1).

### Comparison of diffusion tensor imaging measures

**FIGURE 1:**
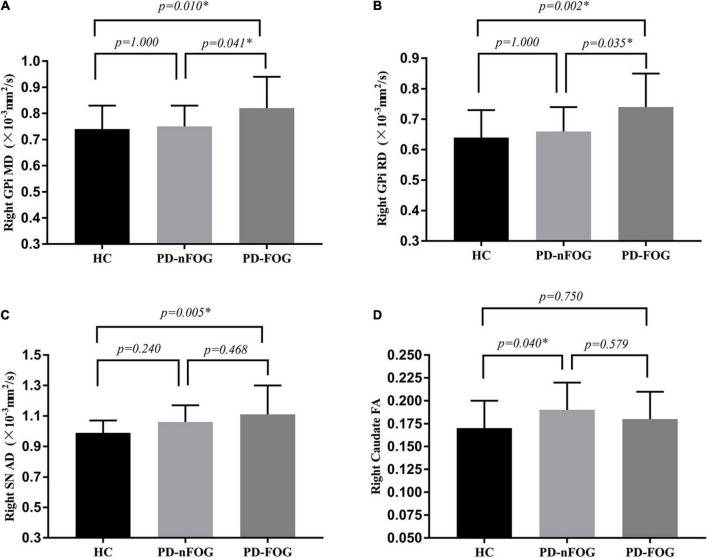
Comparison of DTI Measures across groups. **(A)** MD value of the right GPi; **(B)** RD value of the right GPi; **(C)** AD value of the right SN; **(D)** FA value of the right caudate. *p < 0.05. MD, Mean Diffusivity; RD, radial diffusivity; AD, axial diffusion; FA, fractional anisotropy; GPi, globus pallidus internus; SN, substantia nigra; HC, healthy controls; PD-nFOG, PD patients without FOG; PD-FOG, PD patients with FOG.

Significant differences were observed in MD and RD values of the right GPi among the three groups (MD, *F* = 5.203, *p* = 0.008; RD, *F* = 6.652, *p* = 0.002). Pairwise comparisons showed that the MD value was significantly higher in patients with PD-FOG compared to PD-nFOG (*p* = 0.041) and HC groups (*p* = 0.010, Figure 1A). Higher RD value was also found in PD-FOG patients as compared to PD-nFOG patients (*p* = 0.033) and HC (*p* = 0.002, Figure 1B). No significant differences were seen in FA and AD values of the right GPi among PD-FOG, PD-nFOG, and HC groups.

The one-way ANOVA testing showed significant differences in the AD value of the right SN (*F* = 3.226, *p* = 0.046). The AD value of the right SN was significantly higher in patients with PD-FOG compared with the HC group (*p* = 0.005, Figure 1C). No statistical difference in AD value of the right SN between PD-FOG patients and PD-nFOG, or between PD-nFOG and HC group.

Significant differences were also reveled in FA value of bilateral caudate (right caudate: *F* = 3.226, *p* = 0.046; left caudate: *F* = 3.564, *p* = 0.034). Pairwise comparisons showed that the FA value of the right caudate was significantly higher in patients with PD-nFOG patients compared to HC (*p* = 0.040, Figure 1D). No significant difference was shown in pairwise comparisons in left caudate.

Detailed DTI measures (MD, RD, AD, FA) of bilateral caudate, GPe, GPi, putamen, thalamus, and SN are shown in Supplementary Table 2.

### Diffusion tensor imaging measures correlations with gait measures

**FIGURE 2:**
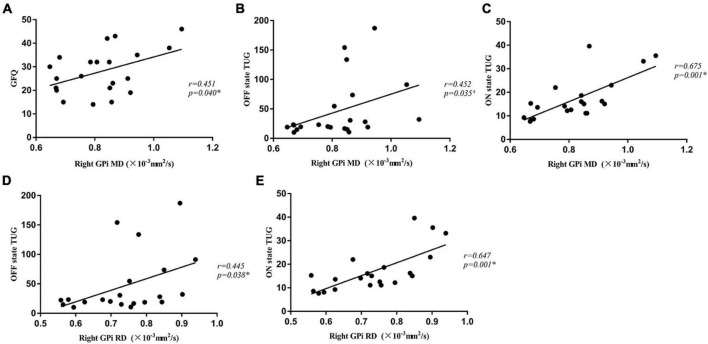
The MD value of the right GPi was positively correlated with GFQ scores **(A)**, times of OFF state TUG **(B)**, and times of ON state TUG **(C)** in the PD-FOG group. The RD value of the right GPi was positively correlated with times of OFF state TUG **(D)** and ON state TUG **(E)** in the PD-FOG group. MD, Mean Diffusivity; RD, radial diffusivity; GFQ, Gait and Falling Questionnaire; TUG, Timed Up and Go tests; GPi, globus pallidus internus.

There were significantly positive correlations between MD values of the right GPi and GFQ scores (*r* = 0.451, *p* = 0.040), and times of OFF state TUG (*r* = 0.452, *p* = 0.035) and ON state TUG (*r* = 0.675, *p* = 0.001) in the PD-FOG group (Figure 2). Significant positive correlations were also shown between RD values of the right GPi and times of OFF state TUG (*r* = 0.445, *p* = 0.038) as well as ON state TUG (r = 0.647, *p* = 0.001) in the PD-FOG group (Figure 2). No significant correlation was found between DTI measures for other nuclei and gait measures.

### Comparison of seed-based resting state functional connectivity

The right GPi was applied as the seed region in the rs-FC analysis as its DTI measures demonstrated significant differences between PD-FOG and PD-nFOG patients, and was correlated with gait measures in PD-FOG patients.

**FIGURE 3:**
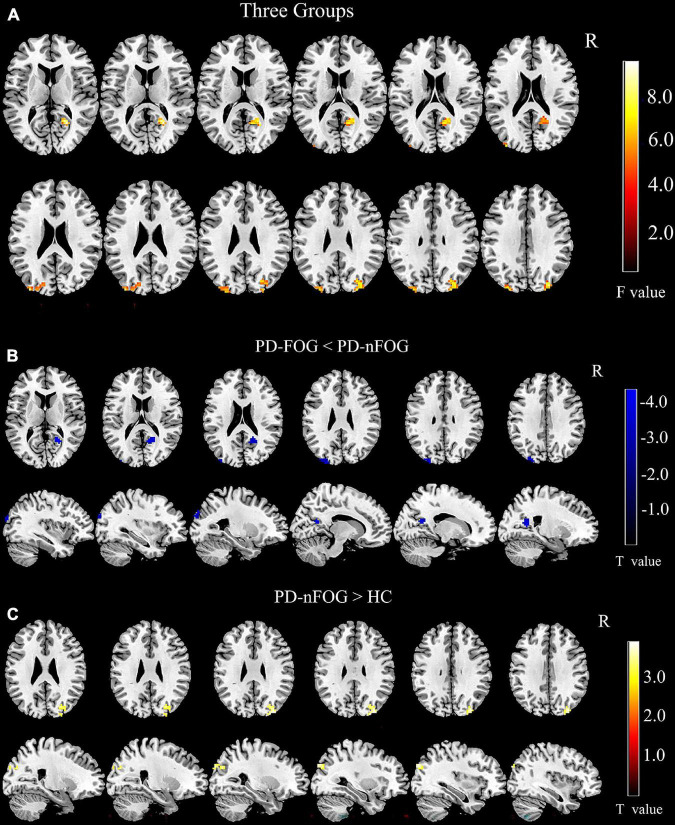
Comparison of Seed-Based Resting State Functional Connectivity. **(A)** Comparison of rs-FC among three groups (voxel threshold *p* < 0.01, clusters AlphaSim corrected *p* < 0.05 and cluster size > 41 voxels); **(B)** Comparison of rs-FC between PD-FOG and PD-nFOG (voxel threshold *p* < 0.01, clusters AlphaSim corrected *p* < 0.01 and cluster size > 33 voxels); **(C)** Comparison of rs-FC between PD-nFOG and HC (voxel threshold *p* < 0.01, clusters AlphaSim corrected *p* < 0.01 and cluster size > 41 voxels).

One-way ANOVA tests showed that the right GPi exhibited different functional connectivity with the right calcarine and bilateral middle occipital gyri among the PD-FOG, PD-nFOG, and HC groups (voxel threshold *p* < 0.01, clusters AlphaSim corrected *p* < 0.05 and cluster size > 41 voxels, Figure 3A and Table 2).

**TABLE 2:** Comparison of functional connectivity between PD-FOG, PD-nFOG, and HC group.

Brain regions	MNI coordinate (mm)			*p*	Cluster size (voxel)
	*x*	*y*	*z*		
**Three Groups**					
Calcarine_R (AAL)	24	–57	12	<0.05	53
Occipital_Mid_L (AAL)	–36	–90	24	<0.05	46
Occipital_Mid_R (AAL)	33	–84	33	<0.05	41
**PD-FOG vs. PD-nFOG**					
Calcarine_R (AAL)	24	–57	12	<0.01	33
Occipital_Mid_L (AAL)	–24	–90	33	<0.01	40
**PD-nFOG vs. HC**					
Occipital_Mid_R (AAL)	33	–87	30	<0.01	41

AAL, automated anatomical labeling; NMI152, Montreal neurological institute 152; HC, healthy controls; PD-nFOG, PD patients without FOG; PD-FOG, PD patients with FOG.

Pairwise comparisons found that PD-FOG exhibited significantly decreased functional connectivity between the right GPi and the left middle occipital gyrus compared to PD-nFOG (voxel threshold *p* < 0.01, clusters AlphaSim corrected *p* < 0.01 and cluster size > 33 voxels, Figure 3B and Table 2). Interestingly, compared with the HCs, PD-nFOG subjects had significantly higher connectivity between the right GPi and the right middle occipital gyrus (voxel threshold *p* < 0.01, clusters AlphaSim corrected *p* < 0.01 and cluster size > 41 voxels, Figure 3C and Table 2). No significant differences were found in rs-FC between the PD-FOG and HC groups.

### Correlations between functional connectivity and gait measures

**FIGURE 4:**
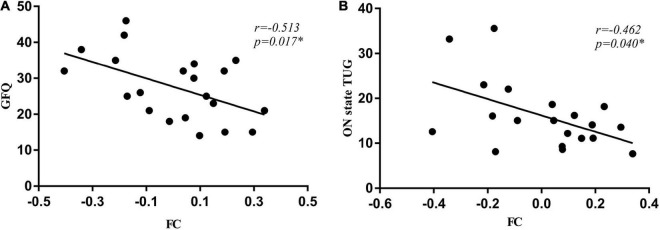
The connectivity between right GPi and left middle occipital gyrus was negatively correlated with the GFQ score **(A)** as well as times of ON state TUG **(B)**. GFQ, Gait and Falling Questionnaire; TUG, Timed Up and Go tests.

In PD-FOG subjects, the connectivity between right GPi and left middle occipital gyrus was negatively correlated with the GFQ score (*r* = –0.513, *p* = 0.017, Figure 4A) as well as ON state TUG (*r* = –0.462, *p* = 0.040, Figure 4B). No significant correlation was detected between the right GPi connectivity to the right calcarine and gait parameters.

## Discussion

The present study applied a multimodal neuroimaging strategy that included DTI and rs-fMRI techniques in the PD-FOG, PD-nFOG, and HC groups. The results demonstrated that the MD and RD values in the right GPi were significantly higher in patients with PD-FOG compared to PD-nFOG and HC, and were positively correlated with the severity of gait dysfunction. Our results also revealed that the FC between the right GPi and the left middle occipital gyrus was reduced in PD-FOG patients compared with PD-nFOG subjects, and it was associated with worse gait performance. These findings suggest the altered microstructure in the GPi and decreased connectivity between the GPi and visual cortex might play a key role in the pathogenesis of PD-FOG.

In our study, higher MD and RD values were detected in PD-FOG patients and positively correlated with FOG severity. The MD value provides a measure of overall diffusivity (Pelizzari et al., 2019), and an increased MD value usually represents broad cellular damage, for example, atrophy, impaired cellularity, edema, or necrosis (Zhang and Burock, 2020). On the other hand, the RD value is determined by the average of the two smaller eigenvalues of water molecule diffusion (Pelizzari et al., 2019), and elevated RD value represents de- or dys-myelination, changes in the axonal diameters or density (Zhang and Burock, 2020). Previous study also observed higher MD and RD values in the globus pallidus in PD patients of the postural instability and gait difficulties (PIGD) subtype compared to controls, and the MD value in the globus pallidus showed a positive correlation with motor severity in the PIGD group (Nagae et al., 2016), in consistency with our finding. Recently, Lench et al. (2022) using diffusion kurtosis imaging approach observed that PD patients with dopa-resistant FOG had higher RD value for the right GPi compared to those with dopa-responsive FOG, further supporting the link between GPi microstructure change and FOG in PD. In conclusion, the MD and RD changes in the right GPi may contribute to FOG in PD and may serve as an imaging biomarker of PD-FOG. However, the postulation needs to be further tested.

Globus pallidus internus is a crucial component of the neural network that is closely linked with FOG. It has been observed that the GPi and substantia nigra *pars reticulata* (SNr) serve as output nuclei of the basal ganglia and provide inhibitory afferent connections to the pedunculopontine nucleus (PPN) and thalamus that control gait (DeLong, 1990). The “cross-talk” model assumes that a considerable loss of dopamine in the striatum in PD patients causes increased firing rate within GPi and SNr GABAergic neurons. Under situations of motor, cognitive, and limbic information overload, GPi and SNr may cause paroxysmal inhibition of the thalamus and PPN, resulting in freezing episodes (Lewis and Barker, 2009; Shine et al., 2013b). In line with our study, several previous neuroimaging studies supported the globus pallidus being involved in the pathogenesis of FOG (Shine et al., 2013a; Peterson et al., 2014; Vercruysse et al., 2014; Mi et al., 2017).

From a network perspective, we found a decreased FC between the GPi and the visual cortex in PD-FOG patients compared with PD-nFOG patients. Motor-visual information transfer plays important role in motor control. It has been demonstrated that visual functional deficit impacted gait impairment in PD (Stuart et al., 2017). Interestingly, the FC between the right GPi and the visual cortex in PD-nFOG was increased compared with PD-FOG and HC. In PD-nFOG patients, visual input may compensate for impaired motor control, which may be sufficient to prevent FOG development. In PD-FOG patients, however, the compensatory mechanism was absent, possibly resulting in the development of FOG. In line with this finding, FOG can be overcome by external visual cueing in clinical observation (Ferraye et al., 2016). Furthermore, previous structural imaging studies revealed severer visual cortical atrophy in PD-FOG patients compared with PD-nFOG patients (Tessitore et al., 2012a; Pietracupa et al., 2018), and a decreased functional connectivity within the visual network (Tessitore et al., 2012b; Canu et al., 2015; Ruan et al., 2020). Theoretically, more severe structural and functional alterations in the GPi-visual cortex may interrupt the integrity of motor-visual information transfer and diminish the compensation by visual input. In the current study, the higher FC between the right GPi and the visual cortex was associated with better gait performance, supporting the compensatory mechanism of GPi-visual cortex connectivity in gait control in PD patients.

Notably, the current study showed alterations mainly in the right hemisphere of PD-FOG patients, which is consistent with a growing body of literature demonstrating that the right hemisphere appears to be selectively affected in FOG (Fling et al., 2013; Peterson et al., 2014; Bharti et al., 2020; Lv et al., 2021; Song et al., 2021; Lench et al., 2022). The laterality of imaging abnormalities in PD-FOG patients is likely related to the fact that the right hemisphere plays a strong role in visuospatial function in right-handed subjects (Joseph, 1988). As mentioned above, the visual function may be a compensatory mechanism. Therefore, those with microstructural damage to the right GPi and dysconnectivity between GPi and the visual cortex may be more susceptible to FOG. Future research should investigate gait performance in predominantly left- or right-side affected PD patients, and assess the relationship between the laterality of symptoms and the asymmetry of imaging changes.

There are some inconsistencies between our observation and previous DTI studies investigating subcortical microstructure change in PD-FOG. Youn et al. (2015) observed a higher MD value in the thalamus in PD-FOG, compared with PD-nFOG patients. However, our study did not find alterations in caudate, putamen, GPe, and thalamus between PD-FOG and PD-nFOG patients. It might be due to variances in the participants involved. Differences in brain atrophy and pathological changes across patients may affect DTI parameters. More studies with larger sample sizes are needed to identify DTI patterns underlying PD-FOG patients. In our finding, there was an increase in the AD value of the right SN in PD-FOG patients compared with HC, but no significant correlation was found between the AD value of the right SN and the severity of FOG. The microstructural change in SN has been rarely investigated between PD-FOG and PD-nFOG patients in previous studies. The role of SN in the pathophysiology of PD-FOG warrants further investigation.

There are some limitations in our study. The present case-control study does not reflect longitudinal alterations in microstructure and FC. In our future study, the collection of follow-up data would provide more valuable information about longitudinal alterations in microstructure and FC. Besides, the relationship between the laterality of FOG and asymmetry of imaging alterations should be investigated. In addition, our relatively small sample size restricted the generalizability of the results and prevented us from performing a study of subtypes of FOG. Further large-sample, multicenter studies are required to confirm the generalizability of the results and investigate the distinct mechanisms between the subtypes of FOG in Parkinson’s disease.

In conclusion, the microstructural abnormalities of the right GPi were observed in PD-FOG patients and were positively associated with the gait measures. The FC between the right GPi and visual cortex was reduced in PD-FOG patients, compared with PD-nFOG patients, and was negatively related to the gait measures as well. Our findings suggest that microstructural alterations in the right GPi and reduced FC between the right GPi and visual cortex maybe associated with the pathological mechanism of FOG in PD patients.

## Data availability statement

The original contributions presented in this study are included in the article/Supplementary material, further inquiries can be directed to the corresponding authors.

## Ethics statement

The studies involving human participants were reviewed and approved by Ethics Committee of Beijing Tiantan Hospital, Capital Medical University, Beijing, China. The patients/participants provided their written informed consent to participate in this study.

## Author contributions

TF and WK designed the study. WK, XW, YZ, JZ, and HHC contributed to data collection and data analysis. WK and XW drafted the manuscript. TF, BS, and HMC revised the manuscript. All authors contributed to the article and approved the submitted version.

## Footnotes

1
www.fmrib.ox.ac.uk/fsl

2
http://www.restfmri.net/forum/RESTplus

3
http://www.fil.ion.ucl.ac.uk/spm
