# Supplementary material for: Freezing of gait in Parkinson’s disease is associated with the microstructural and functional changes of globus pallidus internus
Source: Front Aging Neurosci. 2022 Aug 18;14:975068. doi: 10.3389/fnagi.2022.975068 (PMC9434315; doi:10.3389/fnagi.2022.975068)
Supplement: Supplementary file 1 [file Data_Sheet_1.docx]

Supplementary Material


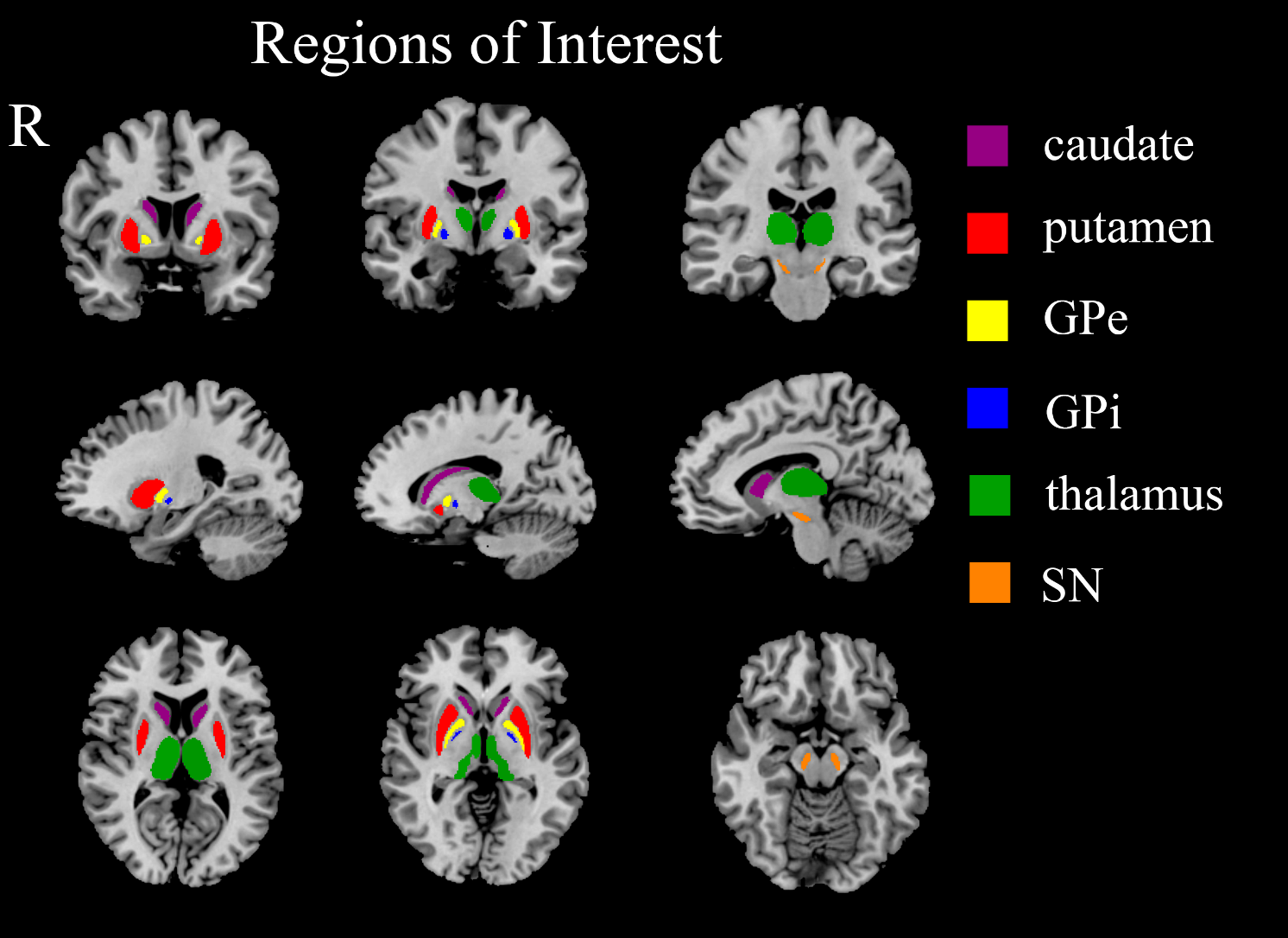


**Supplementary Figure 1** Six regions of interest (ROIs). The ROI masks (probability threshold of 80%) extracted from the Harvard-Oxford atlas or Amsterdam Ultra-high field probabilistic atlas were overlaid on the Colin27 Average Brain using the following color scheme: caudate (violet), putamen (red), globus pallidus externa (GPe; yellow), globus pallidus internus (GPi; blue), thalamus (green), and substantia nigra (SN; orange).

**Supplementary Table 1** Detailed p-values for the Shapiro-Wilk test

|  | PD-FOG | PD-nFOG | HC |
| --- | --- | --- | --- |
| Age | 0.794 | 0.441 | 0.154 |
| Educational years | 0.203 | 0.107 | 0.069 |
| Disease duration | <0.001* | 0.025* |  |
| MDS-UPDRS-III | 0.399 | 0.265 |  |
| LEDD | 0.014* | 0.601 |  |
| MMSE | 0.103 | 0.021* | 0.003* |
| FAB | 0.024* | 0.336 |  |
| HAMA | 0.242 | 0.277 |  |
| HAMD | 0.010* | 0.029* |  |
| SAS | 0.065 | 0.377 |  |

*, *p*＜0.05; H-Y stage, Hoehn-Yahr stage; MDS-UPDRS, Movement Disorder Society Unified Parkinsons Disease Rating Scale; LEDD, levodopa equivalent daily dose; MMSE, Mini-Mental State Examination; FAB, Frontal Assessment Battery; HAMA, Hamilton Anxiety Scale; HAMD, Hamilton Depression Scale; SAS, Starkstein Apathy Scale.

**Supplementary Table 2** Comparison of DTI Measures between PD-FOG, PD-nFOG, and HC group

|  | |  | PD-FOG | PD-nFOG | HC | *p* | *p* value | | |
| --- | --- | --- | --- | --- | --- | --- | --- | --- | --- |
|  |  |  |  |  |  |  | nFOG vs FOG | HC vs FOG | HC vs nFOG |
| **MD value（****×10^-3^mm^2/^s）** | | | | | | | | | |
| caudate | | L | 0.90±0.20 | 0.89±0.23 | 0.91±0.23 | 0.974 | 1.000 | 1.000 | 1.000 |
|  |  | R | 1.05±0.24 | 1.13±0.32 | 1.02±0.22 | 0.344 | 1.000 | 1.000 | 0.454 |
| GPe | | L | 0.60±0.12 | 0.52±0.16 | 0.57±0.16 | 0.171 | 0.185 | 1.000 | 0.814 |
|  |  | R | 0.81±0.09 | 0.77±0.08 | 0.78±0.09 | 0.281 | 0.348 | 0.920 | 1.000 |
| GPi | | L | 0.56±0.20 | 0.5±0.11 | 0.47±0.16 | 0.106 | 0.640 | 0.105 | 1.000 |
|  |  | R | 0.82±0.12 | 0.75±0.08 | 0.74±0.09 | 0.008* | 0.041* | 0.010* | 1.000 |
| putamen | | L | 0.65±0.11 | 0.61±0.11 | 0.62±0.1 | 0.345 | 0.504 | 0.788 | 1.000 |
|  |  | R | 0.79±0.09 | 0.79±0.06 | 0.77±0.07 | 0.512 | 1.000 | 0.848 | 1.000 |
| thalamus | | L | 0.91±0.19 | 0.87±0.12 | 0.87±0.12 | 0.523 | 1.000 | 0.838 | 1.000 |
|  |  | R | 1±0.19 | 0.99±0.13 | 0.96±0.14 | 0.651 | 1.000 | 1.000 | 1.000 |
| SN | | L | 0.63±0.13 | 0.64±0.08 | 0.61±0.06 | 0.440 | 1.000 | 1.000 | 0.615 |
|  |  | R | 0.72±0.09 | 0.7±0.10 | 0.67±0.06 | 0.103 | 1.000 | 0.130 | 0.387 |
| **RD value（×10^-3^mm^2/^s）** | | | | | | | | | |
| caudate | | L | 0.79±0.17 | 0.77±0.21 | 0.77±0.20 | 0.893 | 1.000 | 1.000 | 1.000 |
|  |  | R | 0.92±0.19 | 1.01±0.31 | 0.9±0.20 | 0.260 | 0.662 | 1.000 | 0.350 |
| GPe | | L | 0.49±0.12 | 0.42±0.16 | 0.45±0.18 | 0.309 | 0.387 | 1.000 | 1.000 |
|  |  | R | 0.71±0.09 | 0.66±0.08 | 0.67±0.1 | 0.300 | 0.407 | 0.769 | 1.000 |
| GPi | | L | 0.48±0.19 | 0.44±0.11 | 0.4±0.120 | 0.197 | 1.000 | 0.221 | 0.996 |
|  |  | R | 0.74±0.11 | 0.66±0.08 | 0.64±0.09 | 0.002* | 0.033* | 0.002* | 1.000 |
| putamen | | L | 0.56±0.10 | 0.52±0.10 | 0.53±0.10 | 0.407 | 0.653 | 0.827 | 1.000 |
|  |  | R | 0.72±0.09 | 0.71±0.06 | 0.7±0.07 | 0.640 | 1.000 | 1.000 | 1.000 |
| thalamus | | L | 0.76±0.17 | 0.72±0.11 | 0.71±0.1 | 0.354 | 0.851 | 0.505 | 1.000 |
|  |  | R | 0.83±0.16 | 0.83±0.13 | 0.8±0.1 | 0.562 | 1.000 | 1.000 | 1.000 |
| SN | | L | 0.36±0.11 | 0.35±0.10 | 0.39±0.10 | 0.408 | 1.000 | 1.000 | 0.606 |
|  |  | R | 0.48±0.09 | 0.47±0.13 | 0.47±0.10 | 0.956 | 1.000 | 1.000 | 1.000 |
| **AD value（×10^-3^mm^2/^s）** | | | | | | | | | |
| caudate | | L | 1.14±0.30 | 1.15±0.32 | 1.11±0.27 | 0.840 | 1.000 | 1.000 | 1.000 |
|  |  | R | 1.25±0.30 | 1.38±0.31 | 1.23±0.28 | 0.178 | 0.435 | 1.000 | 0.244 |
| GPe | | L | 0.83±0.13 | 0.75±0.16 | 0.81±0.13 | 0.129 | 0.179 | 1.000 | 0.318 |
|  |  | R | 1.04±0.11 | 0.99±0.09 | 1.03±0.11 | 0.276 | 0.409 | 1.000 | 0.599 |
| GPi | | L | 0.76±0.19 | 0.67±0.11 | 0.66±0.20 | 0.115 | 0.358 | 0.140 | 1.000 |
|  |  | R | 1.00±0.13 | 0.92±0.10 | 0.95±0.13 | 0.092 | 0.093 | 0.488 | 1.000 |
| putamen | | L | 0.83±0.13 | 0.78±0.12 | 0.80±0.11 | 0.404 | 0.578 | 1.000 | 1.000 |
|  |  | R | 0.97±0.11 | 0.96±0.08 | 0.93±0.08 | 0.385 | 1.000 | 0.625 | 0.858 |
| thalamus | | L | 1.2±0.24 | 1.16±0.13 | 1.16±0.15 | 0.657 | 1.000 | 1.000 | 1.000 |
|  |  | R | 1.3±0.24 | 1.29±0.15 | 1.27±0.21 | 0.866 | 1.000 | 1.000 | 1.000 |
| SN | | L | 1.14±0.22 | 1.13±0.19 | 1.02±0.15 | 0.053 | 1.000 | 0.082 | 0.170 |
|  |  | R | 1.11±0.19 | 1.06±0.11 | 0.99±0.08 | 0.006* | 0.468 | 0.005* | 0.240 |
| **FA value** | | | | | | | | | |
| caudate | | L | 0.23±0.04 | 0.27±0.06 | 0.23±0.06 | 0.034* | 0.055 | 1.000 | 0.085 |
|  |  | R | 0.18±0.03 | 0.19±0.03 | 0.17±0.03 | 0.046* | 0.579 | 0.750 | 0.040* |
| GPe | | L | 0.33±0.07 | 0.40±0.12 | 0.35±0.10 | 0.067 | 0.066 | 1.000 | 0.401 |
|  |  | R | 0.24±0.04 | 0.25±0.05 | 0.26±0.05 | 0.280 | 0.828 | 0.357 | 1.000 |
| GPi | | L | 0.35±0.14 | 0.37±0.11 | 0.38±0.10 | 0.586 | 1.000 | 0.911 | 1.000 |
|  |  | R | 0.24±0.04 | 0.24±0.04 | 0.26±0.05 | 0.093 | 1.000 | 0.141 | 0.267 |
| putamen | | L | 0.25±0.06 | 0.28±0.05 | 0.26±0.06 | 0.351 | 0.508 | 1.000 | 0.813 |
|  |  | R | 0.18±0.02 | 0.19±0.02 | 0.18±0.03 | 0.252 | 0.725 | 1.000 | 0.321 |
| thalamus | | L | 0.30±0.02 | 0.31±0.03 | 0.30±0.02 | 0.724 | 1.000 | 1.000 | 1.000 |
|  |  | R | 0.27±0.02 | 0.27±0.02 | 0.28±0.02 | 0.800 | 1.000 | 1.000 | 1.000 |
| SN | | L | 0.68±0.11 | 0.70±0.12 | 0.68±0.11 | 0.863 | 1.000 | 1.000 | 1.000 |
|  |  | R | 0.58±0.09 | 0.61±0.11 | 0.57±0.10 | 0.259 | 0.931 | 1.000 | 0.311 |

*, *p*＜0.05; MD, Mean Diffusivity; RD, radial diffusivity; AD, axial diffusion; FA, fractional anisotropy; HC, healthy controls; PD-nFOG, PD patients without FOG; PD-FOG, PD patients with FOG.
